# Treatment of Dextran Sulfate Sodium-Induced Colitis with Mucosa-Associated Lymphoid Tissue Lymphoma Translocation 1 Inhibitor MI-2 Is Associated with Restoration of Gut Immune Function and the Microbiota

**DOI:** 10.1128/IAI.00091-18

**Published:** 2018-11-20

**Authors:** Kyung Won Lee, Minseok Kim, Chang Hoon Lee

**Affiliations:** a Therapeutics and Biotechnology Division, Center for Information-Based Drug Research Korea and Research Institute of Chemical Technology (KRICT), Daejeon, Republic of Korea; b Department of Animal Science, College of Agriculture and Life Sciences, Chonnam National University, Gwangju, Republic of Korea; c Animal Nutrition and Physiology Team, National Institute of Animal Science, Wanju, Republic of Korea; University of Michigan-Ann Arbor

**Keywords:** MALT1, MI-2, ulcerative colitis, inflammation, microbiome

## Abstract

Disruption of the healthy intestinal microbiome and homeostasis of the intestinal immune system, which are closely interactive, are two key factors for ulcerative colitis. Here, we show that MI-2, a selective inhibitor of mucosa-associated lymphoid tissue lymphoma translocation-1 (MALT1), alleviated excessive inflammatory responses and was associated with restoration of healthy intestinal microbiome in mice suffering from dextran sulfate sodium (DSS)-induced colitis. We found that the diversity of intestinal microbiome of mice with DSS-induced colitis was significantly lower than that of healthy mice. However, MI-2 treatment in mice with DSS-induced colitis resulted in restored microbially diverse populations. To understand the possibility of the beneficial effect of the restored microbially diverse populations of MI-2-treated mice with DSS-induced colitis, we showed that inserting fecal microbiota from MI-2-treated mice with DSS-induced colitis and healthy control mice into mice with DSS-induced colitis could alleviate symptoms of colitis. The possibility of MI-2 treatment in DSS-induced colitis, associated with restoration of healthy microbially diverse populations in addition to reshaping host immune modulating capacity by reducing inflammatory cytokines (tumor necrosis factor alpha, interleukin-1β [IL-1β], IL-17α, and IL-22), may be considered therapeutic for ulcerative colitis.

## Abstract (precis)

Disruption of the healthy intestinal microbiome and homeostasis of the intestinal immune system, which are closely interactive, are two key factors for ulcerative colitis. Here, we show that MI-2, a selective inhibitor of mucosa-associated lymphoid tissue lymphoma translocation-1 (MALT1), alleviated excessive inflammatory responses and was associated with restoration of healthy intestinal microbiome in mice suffering from dextran sulfate sodium (DSS)-induced colitis.

## INTRODUCTION

Ulcerative colitis is a potentially debilitating disease of the gastrointestinal tract that is a well-established risk factor for colorectal cancer (1–3). It affects up to 0.238% of the adult population, thus it represents a major public health burden (4). A limited number of drugs are currently available for colitis treatment. Recent studies have highlighted the role of microbiota in the development of ulcerative colitis, while suppression of colon inflammation was shown to be associated with the preservation of colonic microbially diverse populations and growth of protective commensal bacteria (5, 6). Given that the synergetic effects of the gut microbiome and the host immune system are a critical aspect of ulcerative colitis pathophysiology, treatment with either antibiotics or anti-inflammatory drugs alone is ineffective.

A recent study reported that metformin exerted therapeutic effects in the treatment of type 2 diabetes by altering the intestinal microbiome (7), suggesting that drugs that restore microbiome composition to a normal state can be used to treat diseases. 2-Chloro-N-4-[5-(3,4-dichlorophenyl)-3-(2-methoxyethoxy)-1H-1,2,4-triazol-1-yl]phenylacetamide (MI-2), which was reported as a selective inhibitor for mucosa-associated lymphoid tissue lymphoma translocation 1 (MALT1), displayed little activity against the structurally related caspase family members, caspase-3, -8, and -9 (8). MALT1 is a key regulator of inflammation that is a component of the paracaspase pathway and has proteolytic activity against factors associated with the regulation of immune cell function (9–11).

In this study, we found that inhibition of MALT1 with MI-2 could suppress inflammatory molecules such as tumor necrosis factor alpha (TNF-α), interleukin-1β (IL-1β), IL-17α, and IL-22 in the large intestinal tissues of the *in vivo* mouse model of dextran sulfate sodium (DSS)-induced colitis and alleviate progression of DSS-induced colitis. We also observed significant differences in colon microbiome diversity between mice with colitis and healthy controls, including an increased abundance in the number of Proteobacteria (including Escherichia). Moreover, we found that MI-2-treated mice with DSS-induced colitis reversed the increase in Proteobacteria abundance and recovered colitis-mediated reduction in the phyla Verrucomicrobia and Actinobacteria and genera Akkermansia, Bifidobacterium, and Olsenella. Taking our findings together, we discovered that the therapeutic effect of MI-2 in mice with DSS-induced colitis might be associated with restoration of the balance of the intestinal microbiome and with suppression inflammation, while we could not demonstrate direct evidence of the effect of MI-2 on regulation of intestinal microbial composition. Our results indicate that inhibiting MALT1 is an effective treatment for severe ulcerative colitis.

## RESULTS

### MALT1 inhibition mitigates colitis.

**FIG 1:**
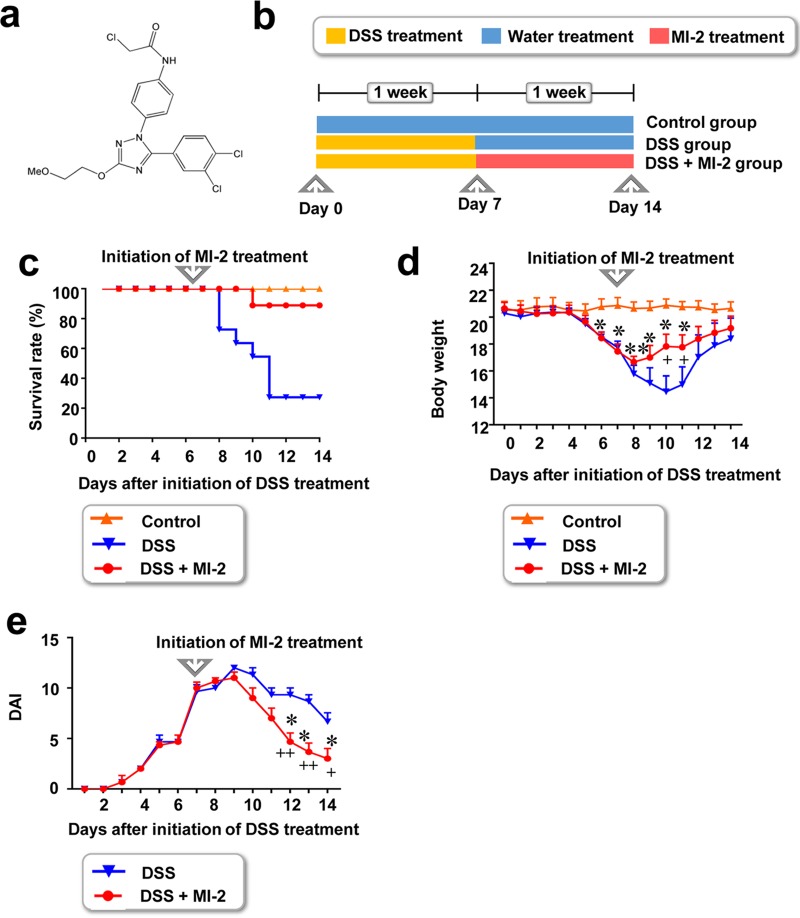
Effect of the MALT1 inhibitor MI-2 in a mouse model of DSS-induced colitis. (a) Chemical structure of MI-2. (b) C57BL/6J mice were administered 3% (wt/vol) DSS in autoclaved drinking water for 7 days to induce colitis, followed by regular autoclaved drinking water for 7 days. Starting from day 7, mice were i.p. injected with MI-2 (25 mg/kg). (c and d) Survival rate and body weight. Results were pooled from three independent experiments (*n* = 10 to 15 mice per group). (e) DAI values calculated based on results pooled from three independent experiments. *P* values were <0.05 (*) and <0.01 (**) between MI-2-treated mice with DSS-induced colitis (DSS + MI-2 group) and untreated healthy mice (control group). *P* values were <0.05 (+) between MI-2-treated and untreated mice with DSS-induced colitis (DSS + MI-2 and DSS groups, respectively) (two-tailed unpaired Student's *t* test). Error bars denote standard errors of the means (SEM).

We evaluated the therapeutic effect of MI-2 (Fig. 1a) in mice with DSS-induced colitis. Mice were first administered 3% (wt/vol) DSS dissolved in drinking water for 7 days, followed by regular water starting on day 8 until the end of the experiment on day 14. Mice in the MI-2 treatment group were given a daily intraperitoneal (i.p.) injection of 30 mg/kg of body weight MI-2 from day 8 to 14, while those in the control group were injected with the same volume of vehicle (180 µl of PBS plus 19 µl of Kolliphor EL and 1 µl of ethanol per mouse) (Fig. 1b). In this study, we wanted to observe the indirect effect of MI-2 on the large intestine microbiome; thus, we avoided oral administration of MI-2, which might affect microbes directly. Instead, we administered an MI-2 compound intraperitoneally. In addition, we tested 10 mg/kg and 30 mg/kg of compound *in vivo* to verify the optimal dose of MI-2 for therapeutic effect. We found that treatment with 10 mg/kg of MI-2 did not significantly alleviate DSS-induced inflammatory bowel disease (IBD). However, treatment with 30 mg/kg of MI-2 exerted a significant effect on mice with DSS-induced IBD (data not shown). Thus, we used a single dose (30 mg/kg) of MI-2 for the *in vivo* experiment.

MI-2-treated mice with DSS-induced colitis showed higher survival rates than untreated mice with colitis (80% versus 20%) (Fig. 1c). Furthermore, a significant recovery in body weight was observed in MI-2-treated mice with colitis compared to that in control animals (Fig. 1d), especially during the most lethal time period (from day 10 to 14) (Fig. 1c). These results indicate that MI-2 treatment mitigates the symptoms of DSS-induced colitis soon after treatment initiation. In addition, mice with colitis that were treated with MI-2 had a lower disease activity index (DAI), which was determined based on intestinal bleeding (12), than their untreated counterparts, starting from day 12 (Fig. 1e).

### MI-2 alleviates damage to intestinal tissue in colitis.

**FIG 2:**
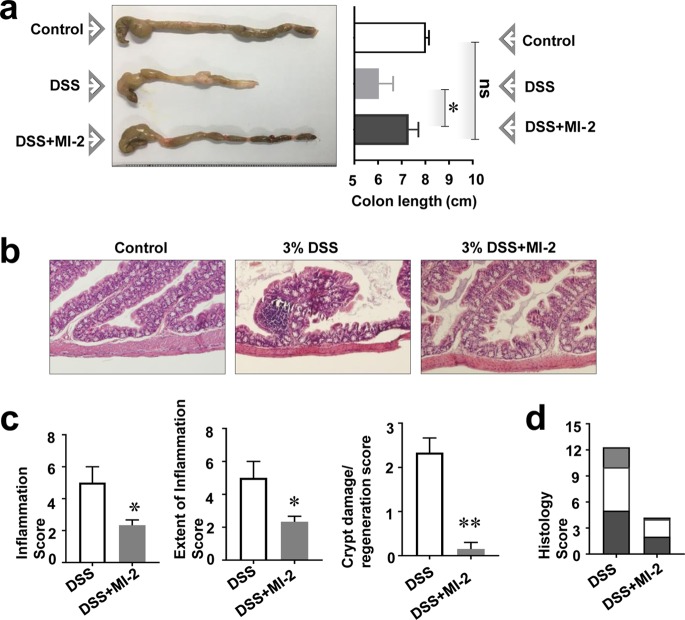
MI-2 restores intestinal tissue damage in DSS-induced colitis. (a) Colon length in control, DSS, and DSS plus MI-2 groups are shown. (Left) Images of colons are representative of three independent experiments (*n* = 10 to 15 mice per group). (Right) The graph shows the mean values ± standard errors of colon lengths pooled from three independent experiments (*n* = 10 to 15 mice per group). (b) Representative images of colon tissue sections stained with H&E from three independent experiments (*n* = 10 to 15 mice per group). (c) Level of crypt damage/regeneration, extent of inflammation, and inflammation score for each sample. Graphs show pooled results from three independent experiments (*n* = 10 to 15 mice per group). (d) Histological scores calculated based on crypt damage/regeneration, extent of inflammation, and inflammation scores (from panel c) for DSS and DSS plus MI-2 mice. *P* values were <0.05 (*) and <0.01 (**) versus control (untreated) cells (two-tailed unpaired Student's *t* test). Error bars denote SEM.

To determine how MI-2 alleviates colitis, we examined the recovery of damaged intestinal tissue. Mice with DSS-induced colitis without MI-2 treatment had a much shorter intestine than control mice; however, MI-2 treatment restored intestinal length to that of healthy animals (Fig. 2a), suggesting that it alleviated tissue deterioration and reduced inflammation associated with colitis. We further evaluated tissue damage in the intestine by analyzing tissue sections stained with hematoxylin and eosin (H&E) and scored according to a previously described system (13). Intestinal tissue from mice with DSS-induced colitis showed evidence of inflammation and crypt damage at multiple sites that were not seen with control mice (Fig. 2b). However, mice with colitis that were treated with MI-2 showed reduced inflammation and crypt damage (Fig. 2b), indicating that DSS-induced intestinal tissue damage was reversed by MI-2 treatment (Fig. 2c and d). To confirm our histological assessment, we measured levels of zona occludens 1 (ZO-1) protein, a barrier protein found in intestinal epithelial cells (14–16), using large intestine tissues of healthy mice with DSS-induced colitis and MI-2-treated mice with DSS-induced colitis. As shown in Fig. S1a and b in the supplemental material, we found that mice with DSS-induced colitis showed significantly reduced ZO-1 protein levels compared to those of healthy mice. Moreover, we found that MI-2 treatment recovered the ZO-1 protein level in mice with DSS-induced colitis (Fig. S1a and b). Because it is well known that an endotoxin molecule, lipopolysaccharide (LPS), is a microbe-derived inflammatory molecule that damages colon epithelial cell monolayers (14, 17), we investigated whether MI-2 could mitigate LPS-induced tissue damage using a Caco2 colon epithelial cell monolayer. To support our hypothesis, we measured the expression of ZO-1 protein in human epithelial cells treated with vehicle, LPS, and LPS plus MI-2 by Western blotting (Fig. S1c and d) and found that the LPS-induced downregulation of ZO-1 was reversed by MI-2.

### MI-2 regulates inflammatory cytokine and antibacterial molecule production by macrophages and intestinal tissue.

**FIG 3:**
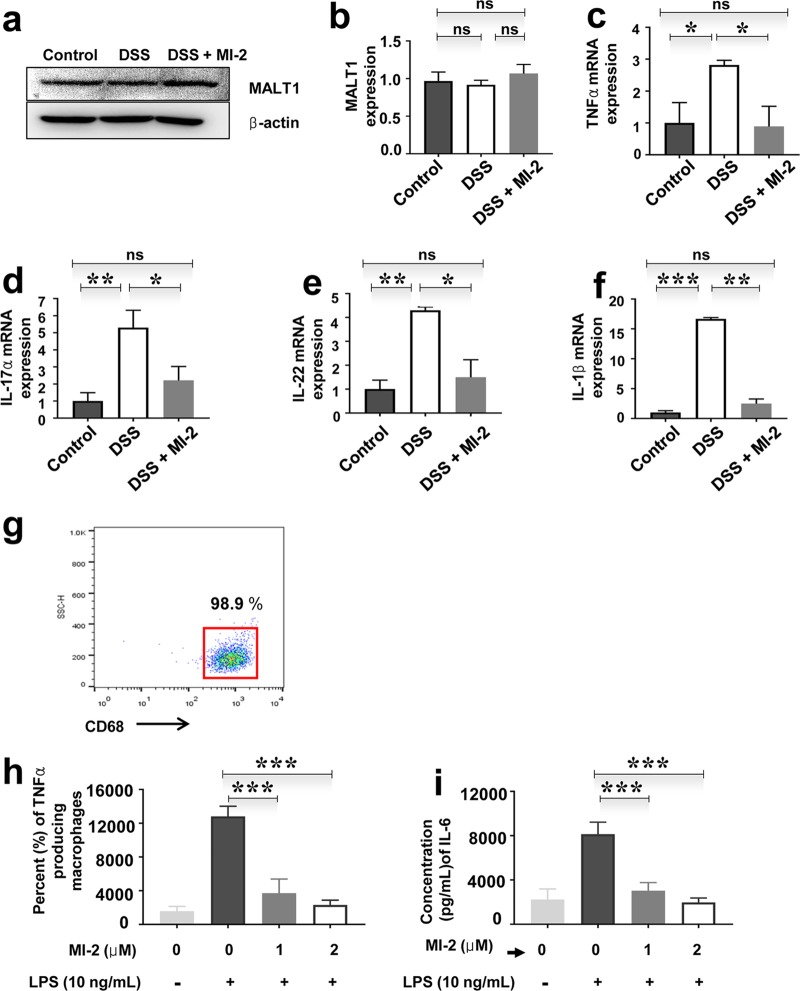
MI-2 inhibits inflammatory cytokine and antibacterial molecule production by macrophages in intestinal tissue. (a) MALT1 protein expression in the colon tissue of control, DSS, and DSS plus MI-2 groups was detected by Western blotting. Images shown are representative of three independent experiments. (b) Quantification of MALT1 protein expression in colon tissue of control, DSS, and DSS plus MI-2 mice based on three independent experiments. (c to f) mRNA expression levels of TNF-α (c), IL-17α (d), IL-22 (e), and IL-1β (f) in colon tissue of control, DSS, and DSS plus MI-2 mice, as detected by qPCR. Graphs are representative of two independent experiments (*n* = 6 per group). (g) Representative dot plot showing the percentage of cells positive for CD68 from three independent experiments. Representative histogram of macrophages differentiated from human peripheral blood monocytes in the presence of M-CSF is shown. Data represent the results of three independent experiments. (h) TNF-α concentration in culture supernatant of untreated or LPS-treated human peripheral monocyte-derived macrophage cultures with or without MI-2 treatment, as determined by ELISA; data are from three independent experiments. (i) IL-6 concentration in culture supernatant of untreated or LPS-treated human peripheral monocyte-derived macrophage cultures with or without MI-2 treatment, as determined by ELISA. Pooled results from three independent experiments are shown. *P* values were <0.01 (**) and <0.001 (***) versus the control group (two-tailed unpaired Student's *t* test). Error bars denote SEM.

MALT1 was expressed in the intestinal tissue of control mice and mice with DSS-induced colitis (Fig. 3a and b). Since cytokines play a critical role in ulcerative colitis, we examined the effect of MALT1 knockdown combined with MI-2 treatment on the expression of cytokines, including TNF-α and IL-17α, -22, and -1β, in intestinal tissue. The transcript levels of all these factors were downregulated by MI-2 treatment in mice with DSS-induced colitis (Fig. 3c to f). We next examined whether MI-2 treatment affects macrophages, an important inflammatory cell type in ulcerative colitis (16). Human macrophages were differentiated from peripheral blood monocytes in the presence of macrophage colony-stimulating factor (M-CSF), and the expression level of the human macrophage marker CD68 was evaluated (Fig. 3g). MI-2 inhibited TNF-α (Fig. 3h) and IL-6 (Fig. 3i) secretion by LPS-stimulated human macrophages. To clarify the mechanism underlying this effect, we knocked down MALT1 expression with a short interfering RNA (siRNA). MALT1 silencing was confirmed by Western blotting (Fig. S2a) and quantitative PCR (qPCR) (Fig. S2b). We found that siRNA-mediated MALT1 knockdown reduced TNF-α and IL-1β levels in LPS-stimulated human macrophages (Fig. S2c and d).

### MI-2 treatment in DSS-induced colitis is associated with restoration of intestinal microbiome diversity in colitis.

**FIG 4:**
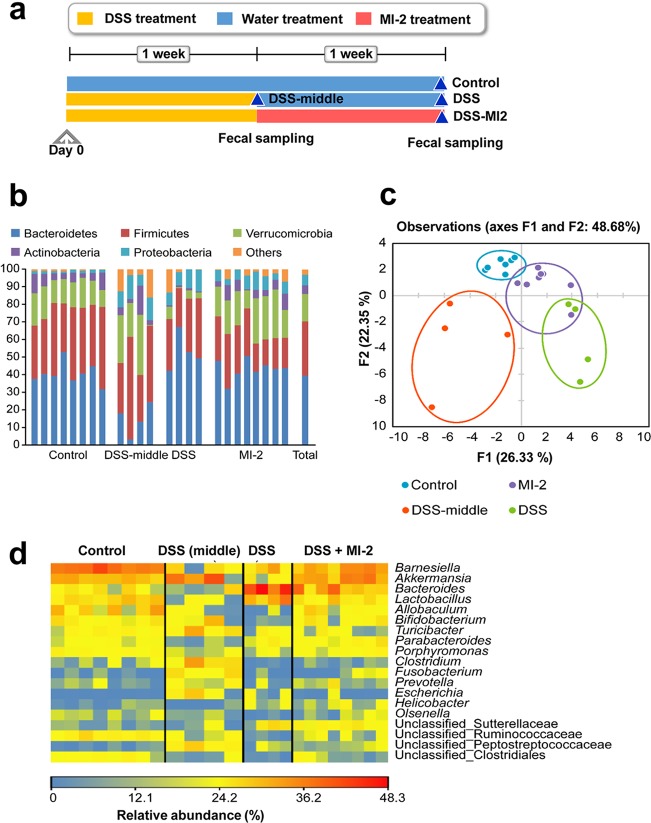
Altered intestinal microbiome composition in MI-2-treated mice with DSS-induced colitis. (a) Microbiome profiles of control, DSS-middle, DSS, and DSS plus MI-2 mice. (b) Relative abundance of phyla in each group. Phyla accounting for <5% of total sequences were combined as Others. Total is the relative abundance of total sequences across all 24 samples in the four groups. (c) PCA of intestinal microbiota. Forty-one OTUs accounting for at least 0.5% of total sequences across all 24 samples in the four groups were included in the analysis. (d) Heat map showing the relative abundance of key bacteria. Genera and unclassified groups accounting for >0.5% of total sequences and differing significantly among the four groups were regarded as key bacteria.

Cytokines such as IL-17α and -22 are known for their antimicrobial function (18, 19). We observed that defensin, an antimicrobial molecule, was downregulated in mice with DSS-induced colitis, an effect that was reversed by MI-2 treatment (data not shown). Since microbiota in the intestine are affected by the host immune system, we speculated that intestinal microbiome profiles differ among control mice and mice with DSS-induced colitis with or without MI-2 treatment. To evaluate this possibility, mice were treated with 3% (wt/vol) DSS dissolved in drinking water for 7 days, followed by regular water on day 7 until the end of the experiment on day 14. Mice in the MI-2 treatment group received daily i.p. injection of 30 mg/kg MI-2, while control animals were injected with the same volume of vehicle. Feces samples harvested from the intestine of mice at designated times were analyzed by 16S rRNA gene sequencing. A total of 909,530 sequences were obtained from 24 samples, with individual samples containing 15,076 to 56,941 sequences. Among the 13 phyla represented by the sequences, Bacteroidetes (39.3%) and Firmicutes (31.1%) were predominant (Fig. 4b), followed by Verrucomicrobia (15.5%), Proteobacteria (5.9%), and Actinobacteria (5.0%). The remaining eight phyla accounted for 3.2% of total sequences (Fig. 4b). The sequences were clustered into 627 operational taxonomic units (OTUs) at 97% sequence similarity, with 41 OTUs accounting for at least 0.5% of all sequences. Alpha diversity indices, including observed OTUs and Chao1 and Shannon’s diversity indexes, were lower in the DSS group than in control animals (*P* < 0.05) (Table 1); however, the diversity was restored to a normal level by MI-2 treatment. The control and MI-2 groups were separated from the other two groups by a second factor (22.35%) in the principal component analysis (PCA) of the 41 OTUs, accounting for at least 0.5% of total sequences in fecal samples and indicating that they harbored similar bacterial communities that differed between the MI-2-treated and untreated groups (Fig. 4c).

**TABLE 1:** Alpha diversity statistics among the four groups

Diet (no. of mice)	Sampling type^*a*^	No. of clean sequences	No. of observed OTUs^*b*^	Chao1 diversity index^*b*^	Shannon diversity index^*b*^
Control (8)	Subsampled reads	15,000	221 ± 19A	276 ± 24A	5.19 ± 0.18A
MI-2 (8)	Subsampled reads	15,000	207 ± 19A,B	278 ± 24A	4.83 ± 0.18A,B
DSS-middle (4)	Subsampled reads	15,000	252 ± 27A	314 ± 35A	4.60 ± 0.26A,B
DSS (4)	Subsampled reads	15,000	138 ± 27B	177 ± 35B	4.39 ± 0.26B

a Means among the four diet groups were compared by ANOVA followed by Duncan’s test.

b Within a column, means with different letters indicate diet groups showing a significant difference (*P* < 0.05). The number of observed OTUs was normalized by randomly subsampling 15,000 clean sequences from each sample.

### MI-2 treatment in DSS-induced colitis is association with alteration of the abundance of specific bacterial taxa.

Verrucomicrobia and Actinobacteria phyla were less abundant in the DSS than in the control and MI-2 groups (*P* < 0.05) (Fig. 4d). Accordingly, the genera Akkermansia (Verrucomicrobia) and Bifidobacterium and Olsenella (Actinobacteria) were less abundant in the DSS than the other three groups (*P* < 0.05) (Fig. 4d). In addition, Allobaculum and Clostridium within Firmicutes and Porphyromonas within Bacteroidetes were less prevalent in the DSS group than in the control and MI-2 groups (*P* < 0.05) (Fig. 4d). On the other hand, Proteobacteria and the constituent genus Escherichia were more abundant in colitis mice without MI-2 treatment than in the other two groups (*P* < 0.05) (Fig. 4d). Taken together, the abundance of the above-described taxa is reduced in mice with DSS-induced colitis but is restored to a normal level in the MI-2-treated mice with DSS-induced colitis. These results indicate that the restoration of abundance of the above-described taxa is associated with MI-2 treatment, although there was no direct evidence to explain how the restoration was triggered by MI-2.

### Transplanted fecal microbiota of MI-2-treated colitis mice alleviates DSS-induced colitis.

**FIG 5:**
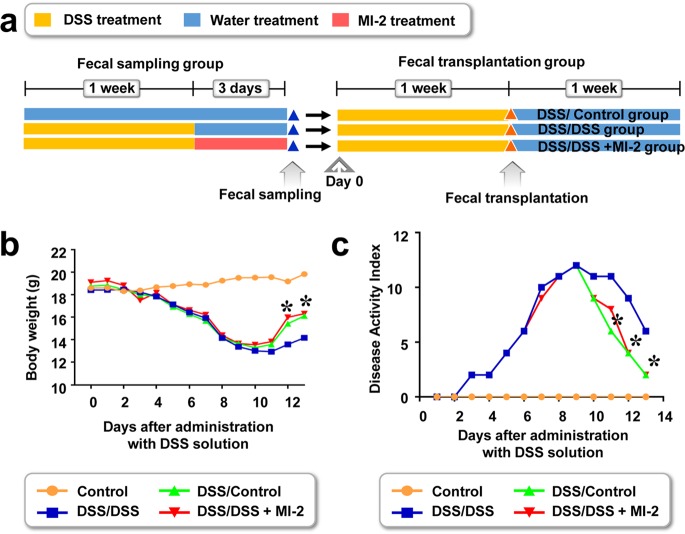
Transplanted fecal microbiota from MI-2-treated mice with DSS-induced colitis and healthy controls increases survival and body weight in mice with DSS-induced colitis. (a) Fecal transplantation from healthy control mice (DSS/Control), mice with DSS-induced colitis (DSS/DSS), and MI-2-treated mice with DSS-induced colitis (DSS/DSS + MI-2) into mice with DSS-induced colitis was performed. (b and c) Body weight was measured (b) and DAI was determined (c), and *r* results were pooled from two independent experiments (*n* = 10 to 12 mice per group). *, *P* value of <0.05 between mice with fecal transplantation from MI-2-treated DSS-induced control mice into mice with DSS-induced colitis (DSS/DSS-MI2) and mice with fecal transplantation from control mice into mice with DSS-induced colitis (DSS/Control). +, *P* value of <0.05 between mice with fecal transplantation from MI-2-treated DSS-induced control mice into mice with DSS-induced colitis (DSS/DSS + MI-2) and mice with fecal transplantation from untreated DSS-induced colitis into mice with DSS-induced colitis (DSS/DSS) (two-tailed unpaired Student's *t* test). Error bars denote SEM.

The intestinal microbiome of healthy mice was more diverse than that of mice with DSS-induced colitis. MI-2 treatment resulted in a near-normal intestinal microbiome in mice with colitis. Thus, a healthy balance of gut bacteria may suppress colitis. To evaluate this possibility, we transplanted intestinal fecal microbiota from MI-2-treated mice with DSS-induced and healthy control mice into mice with colitis (Fig. 5a). Fecal samples were collected on day 10, resuspended in autoclaved PBS containing 10% glycerol, and kept at −80°C until transplantation. DSS-induced colitis was induced and mice were orally administered the fecal solution 7 days later. Colitis mice treated with fecal solution from MI-2-treated and healthy control mice showed greater recovery of body weight (Fig. 5b) and lower DAI (Fig. 5c) than their untreated counterparts. In contrast, fecal solution from mice with DSS-induced colitis failed to improve body weight (Fig. 5b) or DAI (Fig. 5c). These results indicate that the intestinal microbiome in the colon of mice with DSS-induced colitis was changed into a healthier intestinal microbiome upon treatment with MI-2, with alleviation of DSS-induced colitis.

## DISCUSSION

The results of this study show that the MALT1 inhibitor MI-2 mitigated DSS-induced colitis by modulating the host inflammatory response and might be associated with changes in microbiome profile. Interestingly, colitis reduced the diversity of intestinal microbiota, but this was restored in the MI-2-treated mice with DSS-induced colitis. Furthermore, mice with DSS-induced colitis showed an increase in the abundance of Proteobacteria, including Escherichia, relative to that in control mice, which was reversed in the MI-2-treated mice with DSS-induced colitis. Given that bacterial species within an ecosystem compete for resources (20), the observed decrease in gut microbiome diversity in the DSS group might lead to enhanced survival of pathogenic Escherichia coli. Other pathogenic Proteobacteria may also have contributed to the lower diversity, given their greater abundance in mice with colitis. Indeed, the abundance of pathogenic Clostridium difficile, belonging to the phylum Firmicutes, was found to be higher in low-diversity fecal bacterial populations (21). MI-2 treatment was shown to be associated with decreased intestinal pathogens and improved intestinal health in a mouse model of DSS-induced colitis. The abundance of mucin-degrading Akkermansia is reduced in individuals with inflammatory bowel disease (22) and is inversely related to the occurrence of DSS-induced colitis, indicating that Akkermansia colonization has anticolitis effects. In this study, the abundance of the beneficial genus Bifidobacterium, which is known to protect against apoptotic epithelial cell shedding in inflamed intestinal tissue (23), was reduced in mice with DSS-induced colitis but was restored in the MI-2-treated mice with DSS-induced colitis. Interestingly, during the duration of DSS-induced colitis, microbiome profiles were continuously changing. At the middle time point, which we termed the DSS-middle group, we found different microbiome profiles in the DSS group, as shown in Fig. 4b to d. For example, Akkermansia colonization was more abundant in the DSS-middle group than in the DSS-induced mouse group. The beneficial genus Bifidobacterium was more abundant in the DSS group than the DSS-middle group. These changes in microbiome during the duration of DSS-induced colitis may be one of the natural recovery processes in the endogenous protection system of mice. However, as we noted, the final microbial profile of mice that recovered from DSS-induced colitis was still different from that of the healthy mice. For example, DSS-group mice lost Bifidobacterium, unlike healthy mice and even the DSS-middle group mice (Fig. 4d). Since both unclassified Ruminococcaceae and Clostridiales were less prevalent in mice with colitis than in the other groups, unknown members of these families may have anticolitis effects in mice, although additional studies are needed to identify and characterize the actual species. To evaluate the therapeutic effect of changes in microbial profile in the colon of MI-2-treated mice with DSS-induced colitis, we transplanted feces of MI-2-treated mice with DSS-induced colitis into mice with DSS-induced colitis (Fig. 5). In addition to MI-2 restoring the gut immune homeostasis with host immune modulating capacity by reducing inflammatory cytokines (TNF-α, IL-1β, IL-17α, and IL-22), restoration of healthy intestinal microbiome in MI-2-treated mice with DSS-induced colitis may be considered therapeutic for ulcerative colitis.

Moreover, microbial enterotoxins, such as LPS, are microbial inflammatory molecules that damage cell monolayers in the colon (14, 15). LPS production by microbiota might be increased in DSS-induced colitis and aggravates intestinal tissue damage both directly and indirectly by promoting the infiltration of immune cells such as macrophages. More importantly, we showed in this study that ZO-1 protein level in DSS-induced colitis associated with colon tissues of mice was recovered by MI-2 treatment through the intraperitoneal route. MI-2 was previously reported as a marker protein for healthy intestinal barrier (14–16); therefore, this result strongly suggests that MI-2 treatment alleviates colitis-associated intestinal tissue damages. This result was also supported by our histological study shown in Fig. 2b and c. Modulation of immune responses and reestablishment of a healthy gut microbiome balance in the MI-2-treated mice with DSS-induced colitis might promote the recovery of intestinal tissue that is damaged in colitis. Correlation of the onset of inflammation with alterations in the gut microbiota is already well understood (24).

The observed increases in the levels of defensin, TNF-α, and IL-1β, -17α, and -22 in mice with colitis relative to healthy controls were reversed by MI-2 treatment. Excessive inflammation can also damage intestinal tissue and might alter the diversity and composition of intestinal microbiota (25). Excessive levels of TNF-α cause weakening of tight junctions of intestinal epithelial cells, which facilitates the invasion of microbes into host tissues and increases inflammation and tissue damage (25). Our results indicate that MI-2 can restore expression of ZO-1 both *in vitro* and *in vivo* to alleviate inflammation-associated damage to tight junctions (see Fig. S1a and b in the supplemental material).

Indeed, IL-17α and IL-22 are known to regulate microorganisms (18, 19). Although we did not clarify the contribution of the immune response to the modulation of microbiome composition following MI-2 treatment, we speculate that excessive production of inflammatory cytokines affects beneficial microbes, which were then unavailable to control the population of harmful microbes, such as Proteobacteria. Thus, increased proinflammatory cytokines and antibacterial molecule production might exacerbate gut inflammation in colitis, leading to altered intestinal microbiome composition. In conclusion, our results indicate that MI-2 treatment is associated with suppression of inflammation and restoration of microbiome homeostasis in the gut in ulcerative colitis. Thus, selectively inhibiting MALT1 can be an effective therapeutic strategy for the treatment of this disease.

## MATERIALS AND METHODS

### Human peripheral blood collection and cell sorting.

Peripheral blood from healthy donors was obtained from the Red Cross Blood Center (Daejeon, Republic of Korea) according to established guidelines. Methods and protocols used in this study were approved by the Institutional Review Board of the Red Cross, and written, informed consent for study participation was obtained from donors, although donor information was not disclosed. For macrophage differentiation, human monocytes were isolated at >95% purity from peripheral blood based on negative selection using the RosetteSep human monocyte enrichment cocktail (STEMCELL Technologies, Vancouver, Canada). The purity of isolated monocytes was analyzed by flow cytometry using anti-CD14-allophycocyanin (APC) (BioLegend, San Diego, CA, USA), anti-CD16-phycoerythrin-Cy5 (BioLegend), and anti-CD3-APC-Cy7 (BioLegend) antibodies in phosphate-buffered saline (PBS) with 1% fetal bovine serum (FBS) after incubation on ice for 10 min. Data were collected using a FACSCanto II cytometer (BD Biosciences, Franklin Lakes, NJ, USA). Samples were stained with a mixture of the antibodies to set the gates for defining positive and negative cells in multicolor sorting.

### Mouse model of DSS-induced colitis.

Female C57BL/6J mice (6 to 8 weeks old) from Dooyeol Biotech (Daejeon, Republic of Korea) were used for experiments. Mice were housed in a pathogen-free animal facility under a 12-h/12-h light/dark cycle. Acute colitis was induced with 3% (wt/vol) DSS (molecular weight, 36 to 50 kDa; MP Biomedicals, Solon, OH, USA) dissolved in autoclaved drinking water for 7 days. The water was changed every third day. In another set of experiments, mice were induced with DSS until day 7, followed by 3 days of administration of regular water. Mice were sacrificed on day 10 for stool sample collection. Samples from each group of mice were pooled and mixed with sterile PBS, and the supernatant was used for oral administration (1 ml per mouse). Body weight, stool consistency, and occult/gross blood were recorded daily. During the duration of the experiment, a disease activity index (DAI) score was determined to evaluate the clinical progression of colitis. The DAI is the combined score of weight loss compared to initial weight, stool consistency, and bleeding. Weight loss scores were 0 (no loss), 1 (1% to 5%), 2 (5% to 10%), 3 (10% to 20%), and 4 (>20%). Stool consistency scores were 0 (normal), 2 (loose stool), and 4 (diarrhea). Bleeding scores were 0 (no blood), 1 (Hemoccult positive), 2 (Hemoccult positive and visual pellet bleeding), and 4 (gross bleeding, blood around anus) (24). Animal experiments were approved by the Institutional Animal Use and Care Committee of the Korea Research Institute of Bioscience and Biotechnology and were performed in accordance with the *Guide for the Care and Use of Laboratory Animals* published by the U.S. National Institutes of Health (26).

### Reagents.

The MALT1 inhibitor MI-2 (Fig. 1a) was purchased from Sigma-Aldrich (St. Louis, MO, USA) and dissolved in 95% Kolliphor EL and 5% ethanol at a concentration of 30 mg/ml as a stock solution that was stored at −20°C and 10-fold-diluted in PBS before each *in vivo* experiment. The control mice were administered with vehicle solution (180 µl of PBS plus 19 µl of Kolliphor EL and 1 µl of ethanol per mouse). For *in vitro* cell experiments, MI-2 was dissolved in dimethyl sulfoxide (DMSO) at a concentration of 30 mM as a stock solution that was stored at −20°C and optimally diluted in complete culture medium. The final DMSO concentration never exceeded 0.1% in any experiment. Antibodies against MALT1 and actin were obtained from Cell Signaling Technology (Danvers, MA, USA). Enzyme-linked immunosorbent assay (ELISA) kits for human TNF-α were from BioLegend (San Diego, CA, USA). LPS was purchased from InvivoGen (San Diego, CA, USA).

### Generation of monocyte-derived macrophages and measurement of TNF-α and IL-6 levels.

To induce their differentiation into macrophages, isolated human monocytes were cultured in the presence of human recombinant M-CSF (20 ng/ml; Peprotech, Rocky Hill, NJ, USA) in RPMI 1640 medium (Life Technologies, Carlsbad, CA, USA) containing FBS (10%; Life Technologies), l-glutamine (2 mM), and penicillin-streptomycin (Life Technologies) at 37°C and 5% CO_2_. Human recombinant M-CSF was added every 2 days after culture initiation. On day 6, macrophages were treated with LPS (10 ng/ml) and incubated for 18 h in the presence or absence of various concentrations of MI-2. The culture supernatant was collected and maintained at −80°C until analysis, and the TNF-α level in the culture supernatant was measured with the human TNF-α ELISA MAX deluxe kit (BioLegend) according to the manufacturer’s protocol. To identify human macrophages, cells were labeled with antibodies against human CD68 and TNF-α (BioLegend) using the intracellular staining kit (BioLegend) according to the manufacturer’s instructions. Expression data were analyzed on a MACSQuant VYB flow cytometer (Miltenyi Biotec, Bergisch Gladbach, Germany).

### Total RNA isolation and real-time RT-PCR.

Cells were prepared as described above, and total RNA was extracted using TRIzol reagent (Invitrogen, Carlsbad, CA, USA); 20 ng was used as the template for real-time reverse transcription-PCR (RT-PCR) along with qScript cDNA SuperMix for the reverse transcription step and PerfeCTa qPCR FastMix, UNG, ROX for PCR (Quanta Biosciences, Gaithersburg, MD, USA). 6-Carboxyfluorescein/VIC-labeled primer and probe sets were purchased from Applied Biosystems (Foster City, CA, USA). Results were normalized to the level of the glyceraldehyde-3-phosphate dehydrogenase (GAPDH) gene, as detected using TaqMan GAPDH control reagents (Applied Biosystems). Real-time qPCR was performed with duplicate samples using the ABI 7700 Sequence Detection System (Applied Biosystems). For cells from each donor, relative expression levels based on 2^−ΔΔCT^ values are shown as percentages relative to values obtained for the subset with the highest expression.

### H&E staining and histological scoring.

Three longitudinal sections of the colon (proximal, middle, and distal) were fixed with 4% paraformaldehyde, embedded in paraffin in a uniform manner for each mouse, and sectioned at a thickness of 4 μm. The sections were stained with hematoxylin and eosin (H&E) using the appropriate procedures, and dysplasia, crypt damage, and inflammation were described and assessed by a blinded observer according to a previously published protocol (27) for the following measurements: crypt architecture (normal, 0; severe crypt distortion with loss of entire crypts, 3), degree of inflammatory cell infiltration (normal, 0; dense inflammatory infiltrate, 3), muscle thickening (base of crypt sits on the muscularis mucosae, 0; marked muscle thickening present, 3), goblet cell depletion (absent, 0; present, 1), and crypt abscess (absent, 0; present, 1). The histological damage score is the sum of each individual score. Multiple colon sections were stained, and histological scores between similar sections were used to determine the final score for each area (i.e., histological score in proximal colon versus histological score in the distal colon).

### Immunocytochemistry.

Caco-2 cells were seeded on an 8-well μ-slide (ibidi, Planegg, Germany), cultured for 2 days at 37°C and 5% CO_2_, and then ﬁxed with 3.7% paraformaldehyde for 20 min. The slides were washed and permeabilized with PBS containing 0.01% Triton X-100 for 15 min, followed by blocking with PBS containing 1% bovine serum albumin for 1 h at room temperature. The cells were then incubated overnight at 4°C with fluorophore-conjugated primary antibody. The following day, cells were washed three times with 1% bovine serum albumin for 10 min and stained with 4′,6-diamidino-2-phenylindole (DAPI) diluted 1:1,000 in PBS for 10 min. Primary antibody against ZO-1 (Thermo Fisher Scientific, Waltham, MA, USA) diluted 1:200 was used to visualize tight junctions.

### Western blot analysis.

Cells or tissue was prepared as described above and lysed on ice in a buffer composed of 30 mM Tris-HCl (pH 8.0), 75 mM sodium chloride (NaCl), 10% glycerol, and 1% Triton X-100, as well as a protease inhibitor cocktail (Cell Signaling Technology) at 1:100 dilution. Cell or tissue lysates were centrifuged at 12,000 × *g* for 20 min at 4°C and the supernatant was collected. Protein concentration was quantified using the microbicinchoninic acid protein assay kit (Pierce, Rockford, IL, USA) according to the manufacturer’s guidelines, with bovine serum albumin used as a standard. Samples were prepared by boiling at 100°C in 2× Laemmli sample buffer (Bio-Rad) containing β-mercaptoethanol (5%). Cellular proteins (40 µg) were separated by SDS-PAGE at 100 V using a PageRuler Plus prestained protein ladder (Thermo Fisher Scientific) as a marker. After electrophoresis, proteins were transferred onto an Immun-Blot polyvinylidene difluoride membrane (Bio-Rad) for 1 h at room temperature using a Mini Trans-Blot cell (Bio-Rad). The membrane was washed in Tris-buffered saline (20 mM Tris [pH 7.4] and 136 mM NaCl) with 0.1% Tween 20 (TBST), blocked for 1 h in TBST (with 5% nonfat dried milk), and incubated overnight at 4°C in the same solution containing appropriate dilutions of antibodies against human MALT1 and actin (Cell Signaling Technology). Following incubation, the membrane was washed with TBST and incubated for 1 h at room temperature with horseradish peroxidase-conjugated goat anti-mouse or -rabbit antibody (Cell Signaling Technology) diluted 1:10,000 in TBST containing 5% nonfat dried milk. The membrane was washed with TBST at room temperature, and protein bands were visualized using the SuperSignal West Pico chemiluminescent substrate (Pierce).

### Microbiome analysis.

Total community DNA was extracted from fecal samples of 24 mice using the PowerMax soil DNA isolation kit (Qiagen, Valencia, CA, USA). The universal primers 341F and 805R were used to amplify 16S rRNA gene amplicons targeting the V3-V4 region, which were sequenced on the MiSeq platform (Illumina, San Diego, CA). Paired reads were assembled using the FLASH program (28). Sequence processing and microbiome analyses were performed using QIIME v.1.9.1 software (28). The CD-HIT-OTU method (29) was used to remove low-quality and chimeric sequences and calculate OTUs at 97% sequence similarity. The resultant high-quality sequences were classified into taxa using BLASTN v.2.4.0 (29) against the NCBI reference database. The mean proportion of each taxon was log transformed as previously described (30) and compared among the four groups by analysis of variance (ANOVA) followed by Duncan’s test with XLSTAT statistical software (Addinsoft, New York, NY, USA). Alpha diversity in the 24 subsamples was analyzed based on 15,000 sequence reads. OTUs accounting for at least 0.5% of total sequences across all 24 fecal samples were considered major OTUs and were used for PCA with XLSTAT software.

### Statistical analysis.

Multiple comparisons were carried out by ANOVA. Differences between two groups were evaluated with the unpaired *t* test. Statistical analyses were performed using Prism software (GraphPad, Inc., San Diego, CA, USA). A *P* value of <0.05 was considered statistically significant.

## Supplementary Material

Supplemental file 1
