# Supplementary material for: Treatment of Dextran Sulfate Sodium-Induced Colitis with Mucosa-Associated Lymphoid Tissue Lymphoma Translocation 1 Inhibitor MI-2 Is Associated with Restoration of Gut Immune Function and the Microbiota
Source: Infect Immun. 2018 Nov 20;86(12):e00091-18. doi: 10.1128/IAI.00091-18 (PMC6246915; doi:10.1128/IAI.00091-18)
Supplement: Supplemental file 1 [file zii999092602s1.pdf]

Supplementary figure 1: MI-2 treatment reduced the coltis-induced damage on colon tissues *in vovo*.

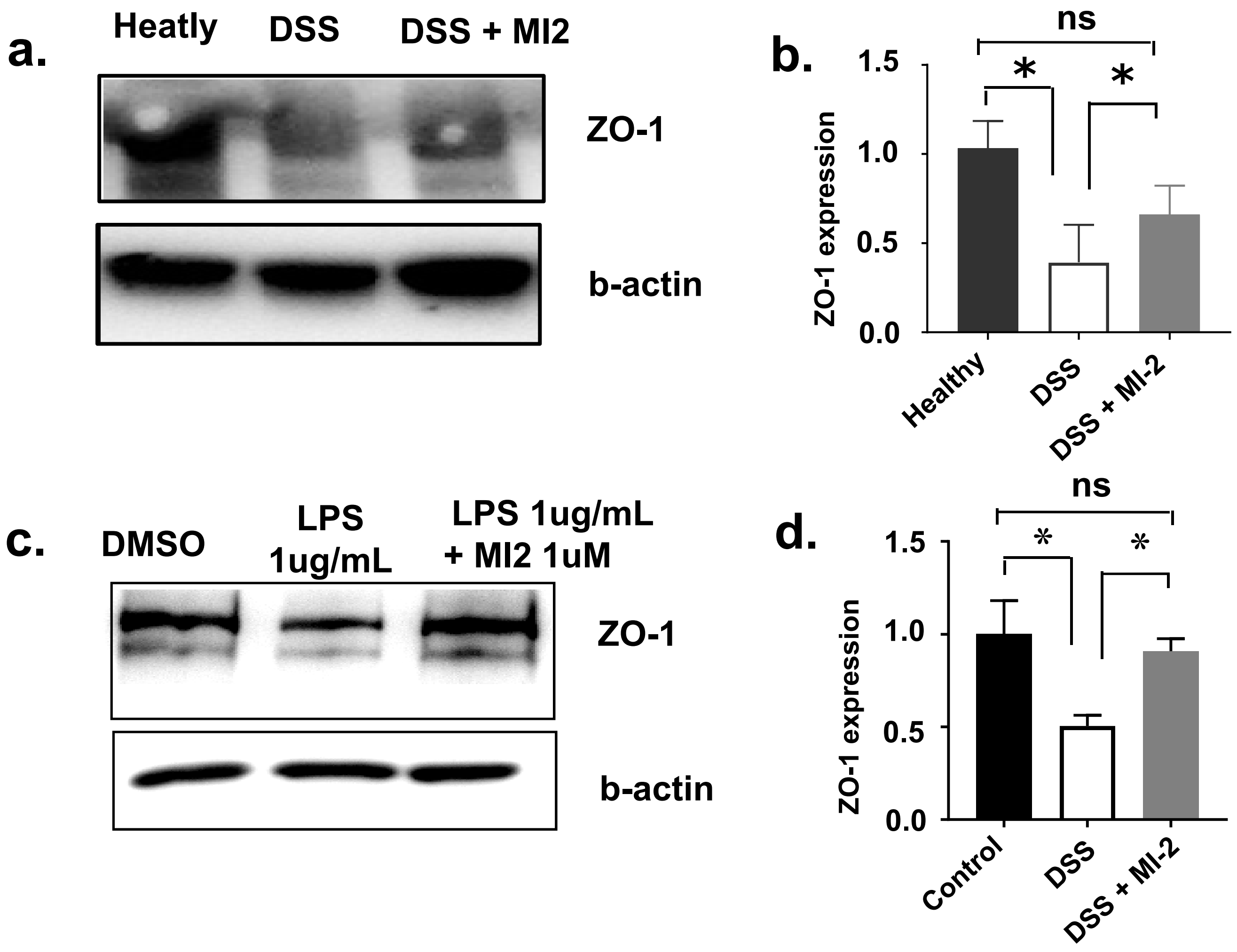

(a) Representative result of western blot analysis shows the expression level of ZO-1 protein from large intestine of healthy or DSS-induced colitis mice with/without treatment of MI-2 (30mg per Kg). Full-lenth blots are presented in Supplementary Figure 3. These results were from three independent experiments. The expression of β-actin was analyzed as a loading control. (b) The graph shows the expression levels of ZO-1 pooled from three independent experiments of large intestine from healthy or DSS-induced colitis mice with/without treatment of MI-2 . (c) Representative result of western blot analysis shows the expression level of ZO-1 protein from Caco2 cell monolayers treated with DMSO, LPS (1 μg/mL), and LPS (1 μg/mL) + MI-2 (2 μM). Full-lenth blots are presented in Supplementary Figure 4. These results were from three independent experiments. The expression of β-actin was analyzed as a loading control. (d) The graph shows the expression levels of ZO-1 pooled from three independent experiments from Caco2 cell monolayers.

Supplementary figure 2 : MALT-1 Knockdown Downregulates the Production of Inflammatory Cytokines TNFα and IL-1β.

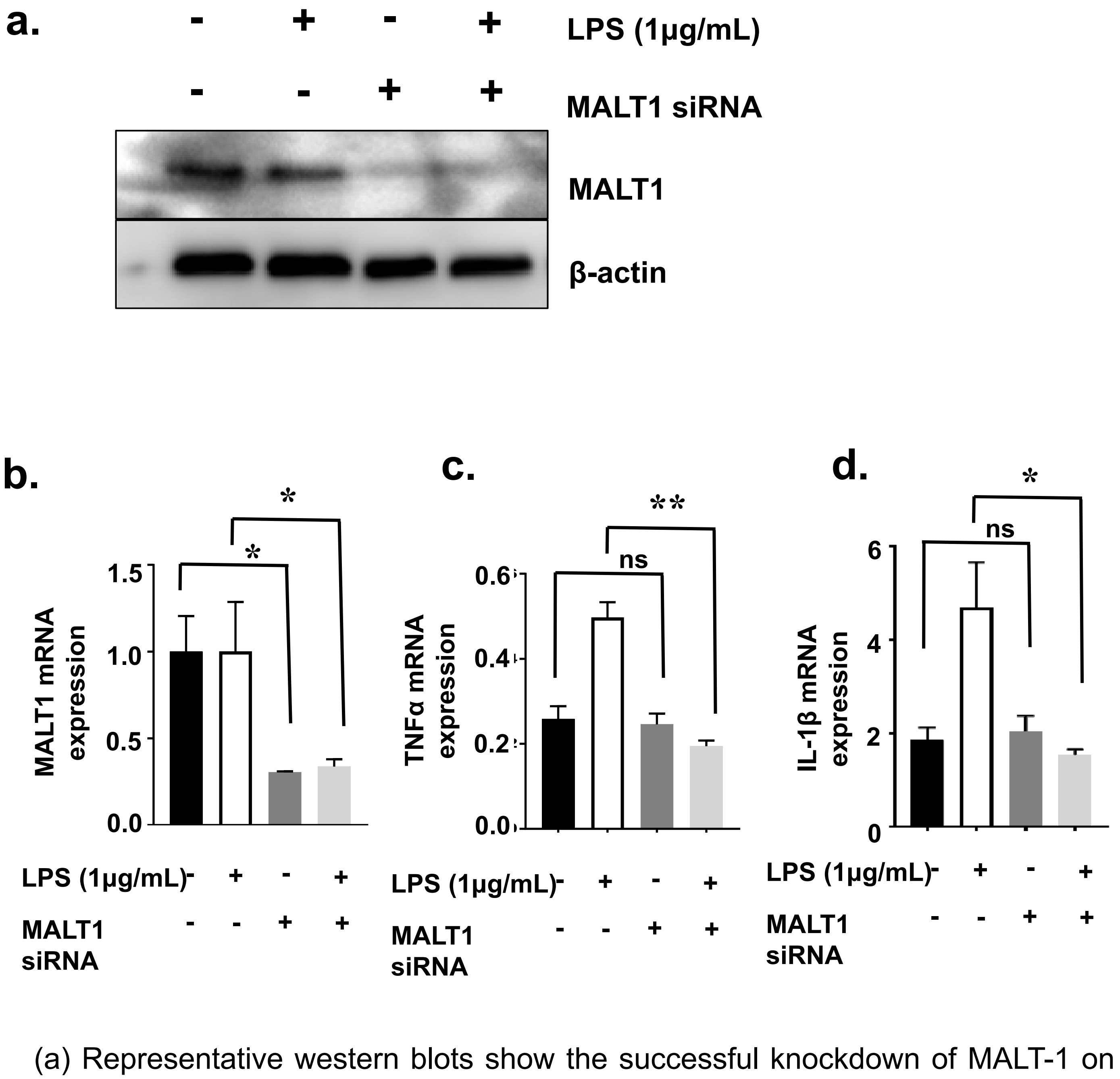

(a) Representative western blots show the successful knockdown of MALT-1 on human macrophages by siRNA from three independent experiments at the protein expression level. Full-lenth blots are presented in Supplementary Figure 4. The mRNA levels of (b) MALT-1, (c) TNFα, and (d) IL-1β were measured from untreated and LPS-treated macrophages with/without MALT-1 siRNA transfection using qPCR method. Pooled results are shown from three independent experiments. ** and *** reflect significant differences based on two-tailed unpaired Student's *t*-tests at $P < 0.01$ and $P < 0.001$, respectively. The error bars show the SEM values.

Supplementary figure 3: Immunoblot analysis of ZO-1 in human epithelial monolayer and mouse colon tissues.

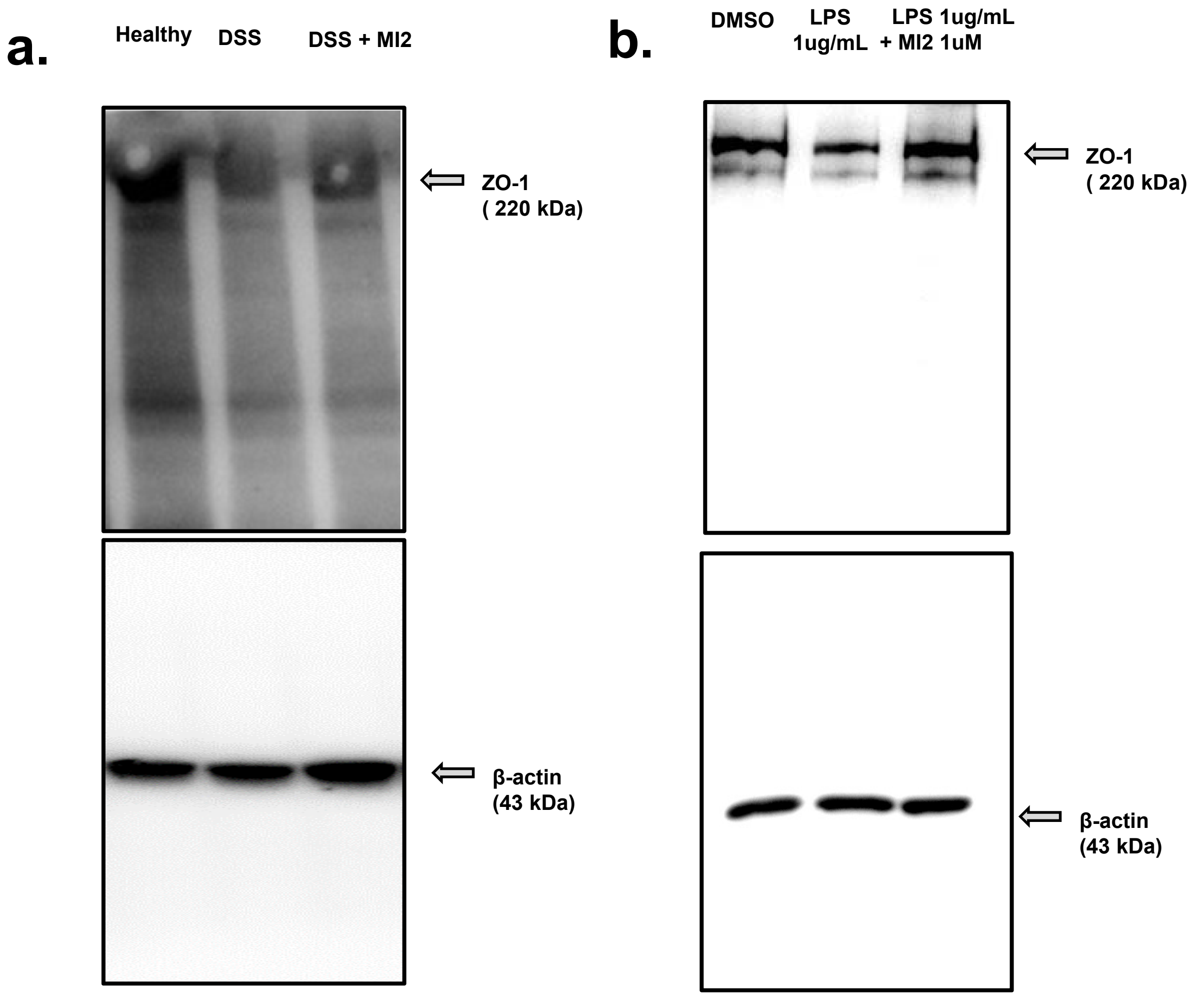

(a) Murine colon tissues were used to assess the expression level of ZO-1 using immunoblot method. Colon tissues of healthy or DSS-induced colitis mice with/without treatment of MI-2 (30mg per Kg) were used for this experiment. (b) Human colon cell line, Caco-2 was used to assesse the expression of ZO-1 using immunoblot method with a use of β –actin as a loading control. Human Caco-2 cells were incubated treated with DMSO, LPS (1 μg/mL), and LPS (1 μg/mL) + MI-2 (2 μM). The level of β-actin was analysed as a loading control.

Supplementary figure 4: Immunoblot analysis of MALT-1 in mouse intestinal tissues and human macrophages.

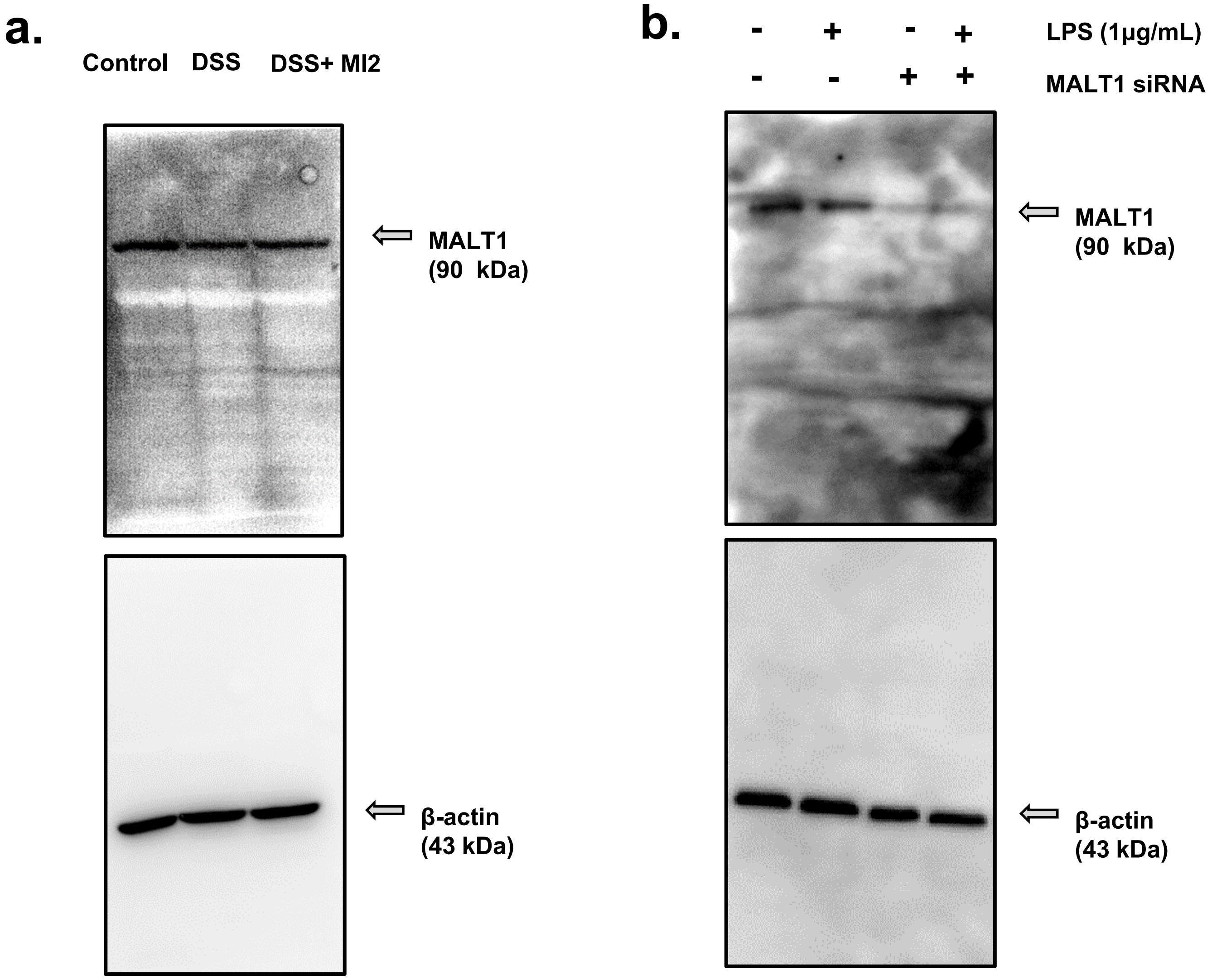

(a) Mouse intestinal tissues of control, DSS and DSS + MI-2 mice group were used to assess the expression of MALT1 using immunoblot method with a use of β –actin as a loading control. N/S indicates non-specific band. (b) Human macrophages derived from peripheral blood monocytes in the absence or presence of LPS with or without knockdown of MALT-1 siRNA were used to assess the expression of MALT-1 using immunoblot method with with a use of β –actin as a loading control.

Supplementary Table. 1 Disease activity index based on previously study

score	Weight loss	Stool consistency	Bleeding stool
0	No weight loss or weight gain	Normal and well formed	Normal color stool
1	5-10 % weight loss	-	-
2	11-15 % weight loss	Very soft and unformed	Reddish color stool
3	16-20 % weight loss	-	-
4	>21 % weight loss	Watery stool	Bloody stool
